# Serotonergic activation during courtship and aggression in the brown anole, *Anolis sagrei*

**DOI:** 10.7717/peerj.3331

**Published:** 2017-05-16

**Authors:** Jacob T. Hartline, Alexandra N. Smith, David Kabelik

**Affiliations:** 1 Department of Biology, Rhodes College, Memphis, TN, United States of America; 2 Program in Neuroscience, Rhodes College, Memphis, TN, United States of America

**Keywords:** Serotonin, Raphe, Reticular nucleus, Aggression, Courtship, Reptile, Fos, Anolis, Lizard

## Abstract

The role of serotonin (5-hydroxytryptamine, 5-HT) in social behavior regulation is not fully understood. While 5-HT release in nuclei of the social behavior network has generally been associated with inhibition of aggressive behavior across multiple classes of vertebrates, less is known about its effects on sexual, especially non-copulatory courtship display behaviors. Furthermore, most research has examined effects at 5-HT release sites, while studies examining the behavioral relevance of source cell populations have generated contradictory findings. This study utilized immunohistochemistry to examine the colocalization of 5-HT with Fos, an immediate early gene product and marker of neural activity, in the raphe and superior reticular nuclei of male brown anoles (*Anolis sagrei*) exposed to either aggression, courtship, or control social interactions. Supporting previous research, copulation was associated with a decrease in 5-HT activity, while a novel link between 5-HT activity and latency to non-copulatory courtship was also found. Within the aggression group, intensity and frequency of behavior were both associated with decreased 5-HT activity. An effect of social context was also seen, with anoles exposed to either courtship or aggression encounters showing decreased 5-HT activity in certain raphe and superior reticular nuclei populations compared to controls. Interestingly, context effects and behavioral effects were seen at separate brain nuclei, suggesting the presence of separate systems with distinct functional roles.

## Introduction

Social behaviors are modulated by various neurotransmitter systems acting on a specific set of brain regions—including the medial amygdala/bed nucleus of the stria terminalis, lateral setpum, preoptic area, anterior hypothalamus, ventromedial hypothalamus, and periaqueductal (central) gray—collectively known as the social behavior neural network; this network has recently been further expanded into a broader social decision-making network by including involved mesolimbic reward system nuclei (Newman, 1999; Goodson, 2005; O’Connell & Hofmann, 2011; O’Connell & Hofmann, 2012). The social behavior network is conserved across multiple classes of vertebrates (Coolen, Peters & Veening, 1998; Crews, 2003; Goodson & Bass, 2002; Sakata et al., 2002; Ball & Balthazart, 2004; Sakata & Crews, 2004; Maney et al., 2008; Teles et al., 2013; Yang & Wilczynski, 2002).

Serotonin (5-HT; 5-hydroxytryptamine) is a neurotransmitter that has been strongly connected to various social behaviors across many classes of vertebrates (Fumero et al., 1994; Larson & Summers, 2001; Rhodes, Yu & Yamaguchi, 2007; Watt et al., 2007; Dennis & Cheng, 2012; Audero et al., 2013; Teles et al., 2013). Serotonin is an evolutionarily conserved molecule (Hay-Schmidt, 2000) that is broadly distributed throughout the brain (Steinbush, 1981). While 5-HT populations in the lateral tegmentum and hypothalamus have also been described in chickens, crocodiles, and some monotremes (Dubé & Parent, 1981; Manger et al., 2002; Rodrigues et al., 2008), serotonin cell body localization in the raphe system and reticular nucleus is conserved across vertebrate classes (Wolters et al., 1985; Bennis et al., 1990; Ayala-Guerrero, Huitrón-Reséndiz & Mancilla, 1991; Stuesse, Cruce & Northcutt, 1991; Jacobs & Azmitia, 1992; Challet et al., 1996). These brainstem 5-HT-producing cells, or source nodes, project to multiple midbrain and forebrain regions, many of which are implicated in social behavior regulation (Kuhar, Roth & Aghajanian, 1971; Lorens & Guldberg, 1974; Hale & Lowry, 2011; Kerman et al., 2011; Kusljic & Van Den Buuse, 2012). Some of these regions, including the amygdala, hippocampus, and nucleus accumbens, have been shown to contain 5-HT receptors and levels of these receptors are predictive of aggression in mice and rats (Nautiyal et al., 2015; Suzuki & Lucas, 2015; Kondaurova et al., 2016). Furthermore, the 5-HT network interacts with other neurotransmitter systems involved in the social behavior network, including dopamine in the striatum, vasopressin in the anterior hypothalamus and medial amygdala, and oxytocin in the raphe nuclei (Messripour & Mesripour, 2013; Morrison & Melloni Jr, 2014; Pagani et al., 2015).

Increasing central 5-HT levels and transmission appears to inhibit aggression in various species, including anoles, birds, and fish (Larson & Summers, 2001; Sperry, Thompson & Wingfield, 2003; Eisenreich & Szalda-Petree, 2015), while receptor antagonism in the hypothalamus and raphe nucleus of hens and mice and decreased central 5-HT signaling in humans have been shown to increase aggression (Jensen et al., 2009; Conner et al., 2010; Dennis & Cheng, 2012; Audero et al., 2013). However, some studies have shown the opposite effects (Bubak, Renner & Swallow, 2014; Shimizu, Kurosawa & Seki, 2016; Stimpson et al., 2016), suggesting a more complex serotonin-aggression paradigm. This complexity may be due to the broad range of involved 5-HT receptors (Olivier, 2015), along with the specifically targeted serotonergic circuitry (Hale & Lowry, 2011). Along with regulating behavior, the 5-HT system appears to respond to conspecific aggressive behavior in the form of decreased levels of released central 5-HT (Van Erp & Miczek, 2000; Korzan & Summers, 2004; Teles et al., 2013), although relatively little work has been done in this area. While 5-HT is linked to consummatory sexual behavior (i.e., ejaculation) (Fumero et al., 1994; Mas, Fumero & González-Mora, 1995; Mehlman et al., 1997), there is currently very little research looking into pre-ejaculatory, courtship behaviors among vertebrates, although some studies suggest that such a relationship may exist (Rhodes, Yu & Yamaguchi, 2007; Pooryasin & Fiala, 2015).

The present study investigates the activation of 5-HT cell populations in the raphe system and superior reticular nucleus of male brown anoles following exposure to male-male aggression and male–female courtship behavioral trials. While existing research has examined the role of 5-HT in social behavior regulation of the green anole (*Anolis carolinensis*) (Korzan et al., 2001; Korzan & Summers, 2004; Watt et al., 2007), no reptile study to our knowledge has examined activity of individual source 5-HT cell populations—as opposed to specific 5-HT release sites—in relation to social encounters. Furthermore, the relationship between 5-HT and appetitive courtship is not well understood, especially in reptiles. While the raphe system is well established in vertebrates, the reticular nuclei are less consistently defined. There is some suggestion that the reticular nuclei of reptiles may be homologous to the mammalian B9 nucleus (Stuesse, Cruce & Northcutt, 1991; Rodrigo-Angulo, Rodriguez-Veiga & Reinoso-Suárez, 2000). However, a pontomesencephalic reticular formation is also known to be present in rats (Hale & Lowry, 2011), and this latter nucleus may also be homologous to the reticular nuclei in lizards. It is even possible that the reticular nuclei of lizards are at least partly homologous with both the B9 nucleus and pontomesencephalic reticular formation, given that both of these latter nuclei share some common functionality (Kerman et al., 2011).

In this study, we perform immunohistochemistry (IHC) to visualize central 5-HT and Fos, an immediate early gene (IEG) product used as a marker of neural activity, in the male brown anole (*Anolis sagrei*), a reptilian species that has successfully been used to study neural networks and social behavior (Kabelik et al., 2013; Kabelik & Magruder, 2014; Kabelik et al., 2014). Reptiles are a phylogenetic class possessing many ancestral characteristics, including brains similar to mammalian ancestors (MacLean, 1978), thereby making reptiles a good comparative, small-scale model of more derived amniote classes.

Based on the large amount of research suggesting an inverse relationship between 5-HT and aggression, we predict exposure to a male-male aggressive social encounter will generally decrease 5-HT cell activation in the male brown anole. Due to a less clear understanding of the relationship between 5-HT and courtship behaviors, no such prediction can be made for the male–female courtship encounter. The results of this study will contribute to the mapping of the reptilian 5-HT neural network and will further elucidate the functional brain-behavior relationship in this species.

## Methods

### Subjects and social condition groups

The subjects examined in this study are the same as those used in previously published vasotocin, mesotocin, and catecholamine studies and detailed methodology can be found in those papers (Kabelik et al., 2013; Kabelik & Magruder, 2014; Kabelik et al., 2014). Fifty-seven adult male brown anole lizards served as the focal subjects. These animals were placed for 72 h into one half of a terrarium (30.5 cm H × 26 cm W × 51 cm L), separated from the other half by an opaque partition. The animals were visually isolated from one another. In the case of the control group (*n* = 12), the other half of the terrarium contained no animal. The other half of the terrarium in the male-male aggression condition group (*n* = 23) contained a conspecific stimulus male up to 2 mm shorter in snout-vent length and the male–female courtship group (*n* = 22) contained a conspecific stimulus female. All procedures involving live animals were approved by the Rhodes College Institutional Animal Care and Use Committee (approval #101-R2).

### Behavioral trials

To commence a trial, the opaque divider was removed and the behaviors of the focal and stimulus animals were observed from behind a blind and scored over a 14-minute interval. Behaviors were scored for frequency as well as intensity; intensity measurements were divided into appetitive display and pursuit behaviors, as well as consummatory biting (in aggression trials) and copulation (in courtship trials). Kabelik et al. (2013) present an ethogram describing the recorded behaviors. At the end of the trial, animals were gently separated and maintained undisturbed for 90 min, at which point they were sacrificed by rapid decapitation. This delay in sacrifice allowed for peak Fos induction, as well as for Fos degradation in cases of reduced neural activity (Herdegen & Leah, 1998).

### Tissue preparation and immunohistochemistry

Immediately following decapitation, brains were dissected and submerged overnight in 4% paraformaldehyde (in 0.1 M phosphate buffer) at 4 °C. Brains were then transferred to 30% sucrose in 0.1 M phosphate-buffered saline (PBS) for another 24 h at 4 °C, frozen and stored at −80 °C. The brains were cryosectioned at 50-μm thickness, and 2 series of alternating coronal sections were kept. The caudal half of series 2 underwent immunohistochemical processing for this study; multiple immunohistochemistry runs were conducted, each containing brains from a mixture of social condition groups. Brains were first rinsed for 30 min in PBS and blocked in a solution of 2.5% donkey serum (Sigma-Aldrich, St. Louis, MO, USA) and 0.03% Triton-X-100 (Fisher Scientific, Waltham, MA, USA) in PBS for 1 h. Sections were then placed for 40 h at 4 °C in the blocking solution containing 0.2 μg/L goat anti-5-HT polyclonal antibody (Immunostar #20079) and 20 μg/L mouse anti-Fos monoclonal antibody (Santa Cruz Biotechnology #sc-166940; Santa Cruz Biotechnology, Dallas, TX, USA). The sections were rinsed again for 30 min in PBS and then incubated at room temperature for 2 h in blocking solution now containing secondary antibodies, 1:500 Alexa Fluor donkey-anti-goat 488 and 1:200 Alexa Fluor donkey-anti-mouse 555 (both from ThermoFisher Scientific, Waltham, MA, USA). Following secondary antibody incubation, sections were rinsed for 30 min in PBS. Sections were then mounted onto gelatin- and chrome-alum-subbed slides, allowed to air dry, and cleared with Xylene Substitute (Sigma-Aldrich, St. Louis, MO, USA) for 5 min. Finally, sections were coverslipped with Prolong Gold mounting medium containing DAPI (ThermoFisher Scientific, Waltham, MA, USA), and allowed to cure for 24 h. Preadsorption with 5x 5-HT blocking peptide (Immunostar, Hudson, WI, USA) eliminated the majority of signal in control tissues, while 25x blocking peptide eliminated all signal.

### Image acquisition and analysis

**Figure 1:**
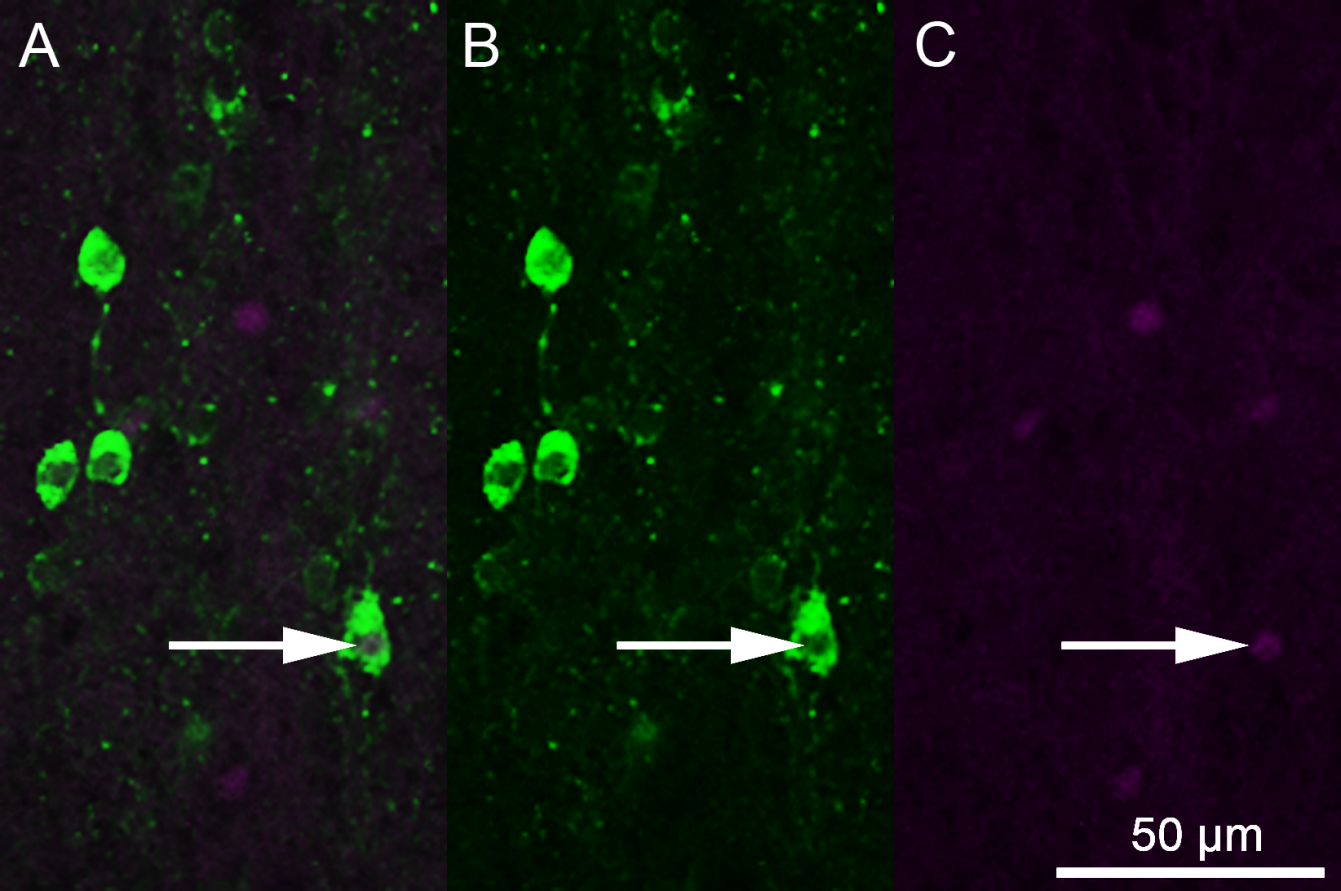
Photomicrographs depicting immunofluorescent labeling of 5-HT (green) and Fos (magenta). (A) A combined image. (B) 5-HT-only image. (C) Fos-only image. The white arrow indicates 5-HT colocalization with Fos.

An LSM 700 confocal microscope was used in combination with Zen 2010 software (Carl Zeiss Inc., Oberkochen, Germany) to acquire fluorescent photomicrographs of 5-HT-ir and Fos-ir neurons. Images were captured using a 20x objective in a z-stack at 10 levels, each 5 μm apart. These images were converted into a two-dimensional projection image and separate colors were exported as separate images using AxioVision 4.8 (Carl Zeiss Inc., Oberkochen, Germany). These images were viewed as stacked monochromatic layers using Adobe Photoshop (Adobe Systems Inc., San Jose, CA, USA). Each layer could be toggled on and off to observe cell and colocalization counts (Fig. 1). A single observer, blind to social condition group, analyzed each brain region. Regional divisions into the superior medial raphe nucleus (RAsm), superior lateral raphe nucleus (RAsl), medial superior reticular nucleus (Rsm), lateral superior reticular nucleus (Rsl), inferior raphe nucleus (RAi), and caudal inferior raphe nucleus (RAic) were developed based on 5-HT mapping in other reptiles (Wolters et al., 1985; Smeets & Steinbusch, 1988; Bennis et al., 1990; Challet et al., 1991; Ayala-Guerrero, Huitrón-Reséndiz & Mancilla, 1991; Kiehn, Rostrup & Møller, 1992; Ten Donkelaar, 1998; Rodrigues et al., 2008). Although not all studies make an explicit medial-lateral division in either of these nuclei, a spatial separation between medial and lateral neuron clusters in both the superior raphe and superior reticular nuclei allowed us to delineate such a division. While no previous subdivision of the inferior raphe has been made to our knowledge, the anole brains in the present study always show two neuron clusters in the inferior raphe region spatially separated by at least one 100-micrometer section of no 5-HT immunofluorescence, thus we have subdivided the region. Serotenergic cells were also seen in other brain regions, but not in sufficient numbers that would allow for analysis in this study.

### Statistical analysis

**Figure 2:**
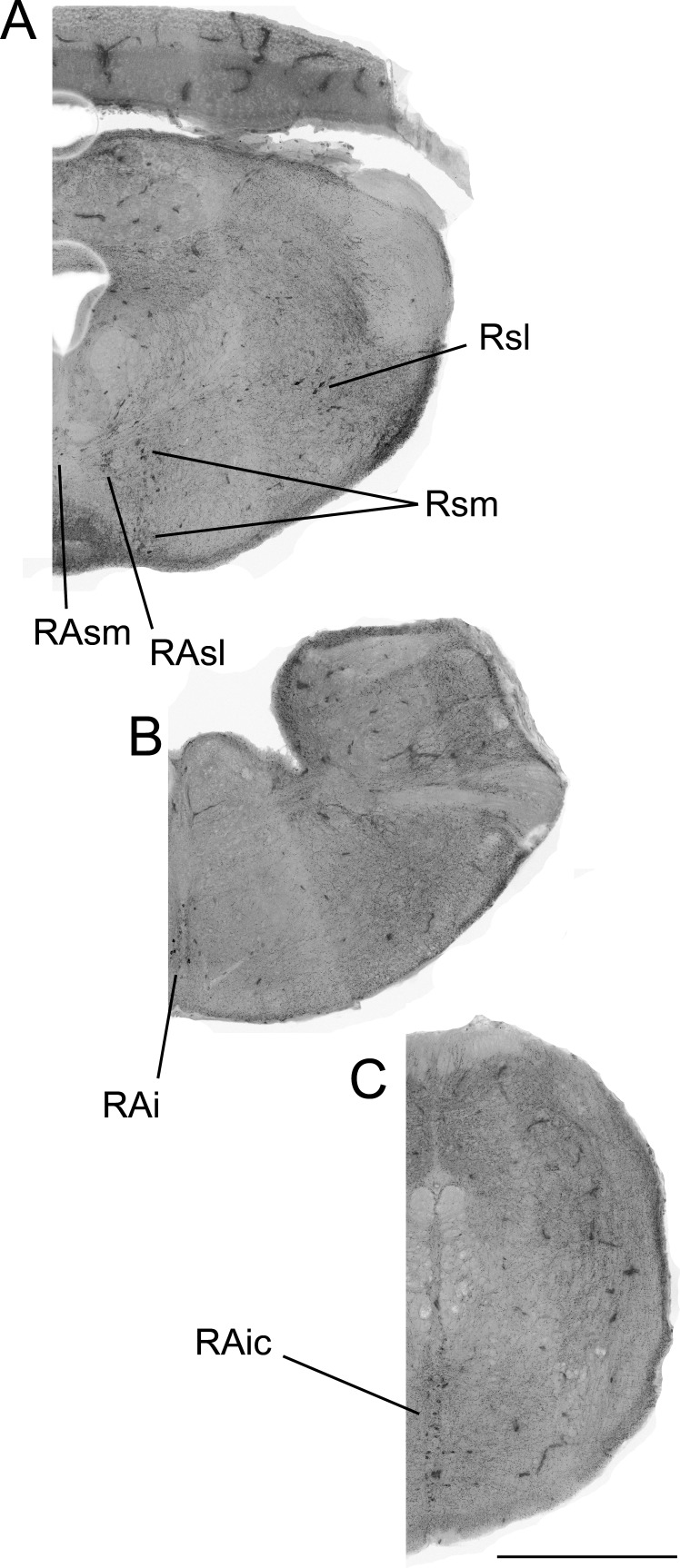
Color-inverted fluorescence photomicrographs representing distribution of 5-HTimmunoreactive neurons from rostral to caudal brainstem regions (A–C). Abbreviations: superior medial raphe nucleus (RAsm), superior lateral raphe nucleus (RAsl), medial superior reticular nucleus (Rsm), lateral superior reticular nucleus (Rsl), inferior raphe nucleus (RAi), caudal inferior raphe nucleus (RAic).

We wished to compare the total number of cells expressing 5-HT-Fos colocalization across social conditions (control, courtship, and aggression), both in the entire brain and in individual brain regions (see Fig. 2 for images of analyzed brain regions). Data from brains containing missing or damaged slides in the region of interest were therefore excluded from this analysis. Because our sample size was reduced by excluding brains with missing or damaged sections, we thus also conducted analyses comparing the percentage of 5-HT cells showing colocalizing with Fos (this relative measure was not as greatly affected by a given missing section). Only brain regions with at least 5 5-HT neurons were included in the percentage analysis.

Some variables did not meet assumptions of parametric analyses so non-parametric tests were employed throughout. In all between-group analyses, non-parametric Kruskal–Wallis tests were conducted, with pair-wise *post hoc* Mann–Whitney tests run at α = 0.05. Mann–Whitney tests were also conducted for analyses of appetitive versus consummatory behavioral intensity. Non-parametric Spearman’s correlations were used to investigate the relationship of behavior frequency and latency within social condition groups with total number of cells expressing 5-HT-Fos colocalization, percentage of 5-HT cells colocalizing Fos, and total number of 5-HT cells. Benjamini–Hochberg corrections to the alpha level were made when correcting for multiple comparisons (Benjamini & Hochberg, 1995).

## Results

### Effects of social condition on 5-HT activity

#### All regions combined

**Figure 3:**
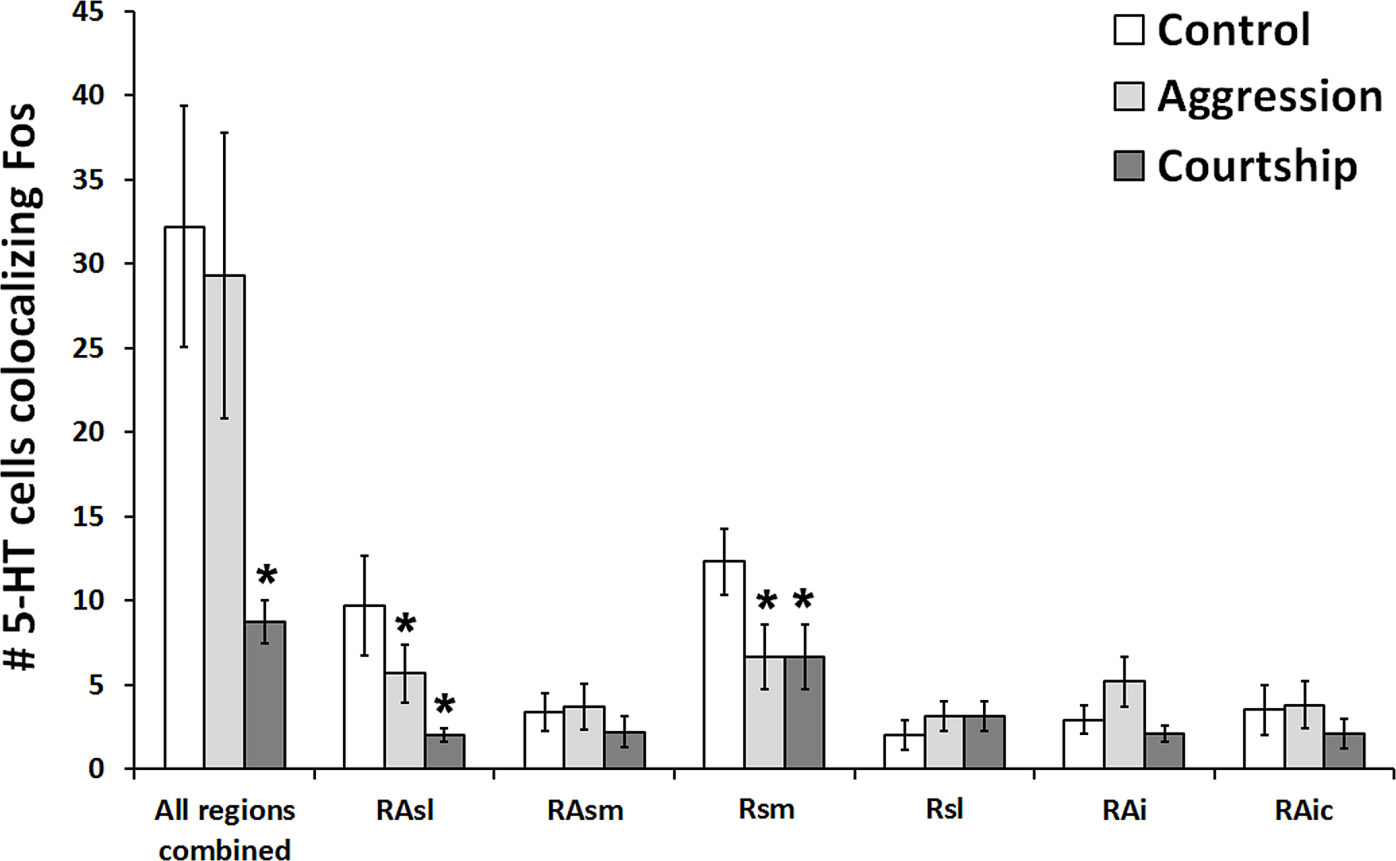
Fos induction in 5-HT neurons across brain regions. Effect of social condition on total number of 5-HT cells colocalizing Fos across multiple brain regions and in all regions combined. (*) indicates significant differences (*p* < 0.05) from control group.

When looking at all of the 5-HT regions together, the number of 5-HT cells colocalizing Fos was found to significantly differ across social conditions (χ^2^ = 7.662, *df* = 2, *p* = 0.022; Fig. 3). Please note that this analysis includes only subjects for whom we had complete datasets (*N* = 38), i.e., no missing sections; however, comparable results were produced even when examining the percentage of 5-HT cells colocalizing Fos (*N* = 57; χ^2^ = 6.907, *df* = 2, *p* = 0.032) rather than the total number of such cells. *Post hoc* pair-wise Mann–Whitney tests indicated that the courtship group (*N* = 14) had significantly fewer 5-HT cells colocalizing Fos (*U* = 24.500, *p* = 0.008) than the control group (*N* = 10) and even marginally fewer counts (*U* = 55.500, *p* = 0.050) than the aggression group (*N* = 14). There were no significant differences between the aggression and control groups in total number of colocalized cells (*U* = 59.000, *p* = 0.519). There was also no difference between groups in total number of 5-HT cells (χ^2^ = 2.194, *df* = 2, *p* = 0.334).

#### Individual regions

When subsequently breaking up our analyses by 5-HT population, we found that 2 out of the 6 examined regions differed across behavioral conditions in the total number of 5-HT cells colocalizing Fos RAsl (χ^2^ = 11.246, *df* = 2, *p* = 0.004) and Rsm (χ^2^ = 11.309, *df* = 2, *p* = 0.004) (Fig. 3). These results both remained significant even if α was adjusted for multiple comparisons using the Benjamini–Hochberg correction. When analyzing percent colocalization, the detected relationships were weaker: RAsl (χ^2^ = 5.430, *df* = 2, *p* = 0.066), Rsm (χ^2^ = 4.945, *df* = 2, *p* = 0.084). In the Rsm, *post hoc* Mann–Whitney tests indicated a reduced number of 5-HT cells colocalizing Fos in both the aggression (*N* = 21) and courtship (*N* = 20) groups compared to the control (*N* = 10) group (*U* = 51.000, *p* = 0.022 and *U* = 25.000, *p* = 0.001 respectively). In the RAsl, *post hoc* tests also revealed a reduced number of 5-HT cells colocalizing Fos in the aggression (*N* = 20) and courtship (*N* = 17) groups compared to the control (*N* = 10) group (*U* = 54.000, *p* = 0.044 and *U* = 13.000, *p* = 0.0001 respectively). No other regions showed significant group differences (*p* > 0.05 for all).

### Relationship between behavior intensity and 5-HT activity

#### All regions combined

When examining all 5-HT regions combined, there were no significant relationships between neural measures of 5-HT colocalization with Fos and behavioral intensities in either courtship or aggression trials (*p* > 0.05 for all).

However, when analyzing brain regions separately, significant relationships were present, as described below.

#### Courtship intensity (individual regions)

**Figure 4:**
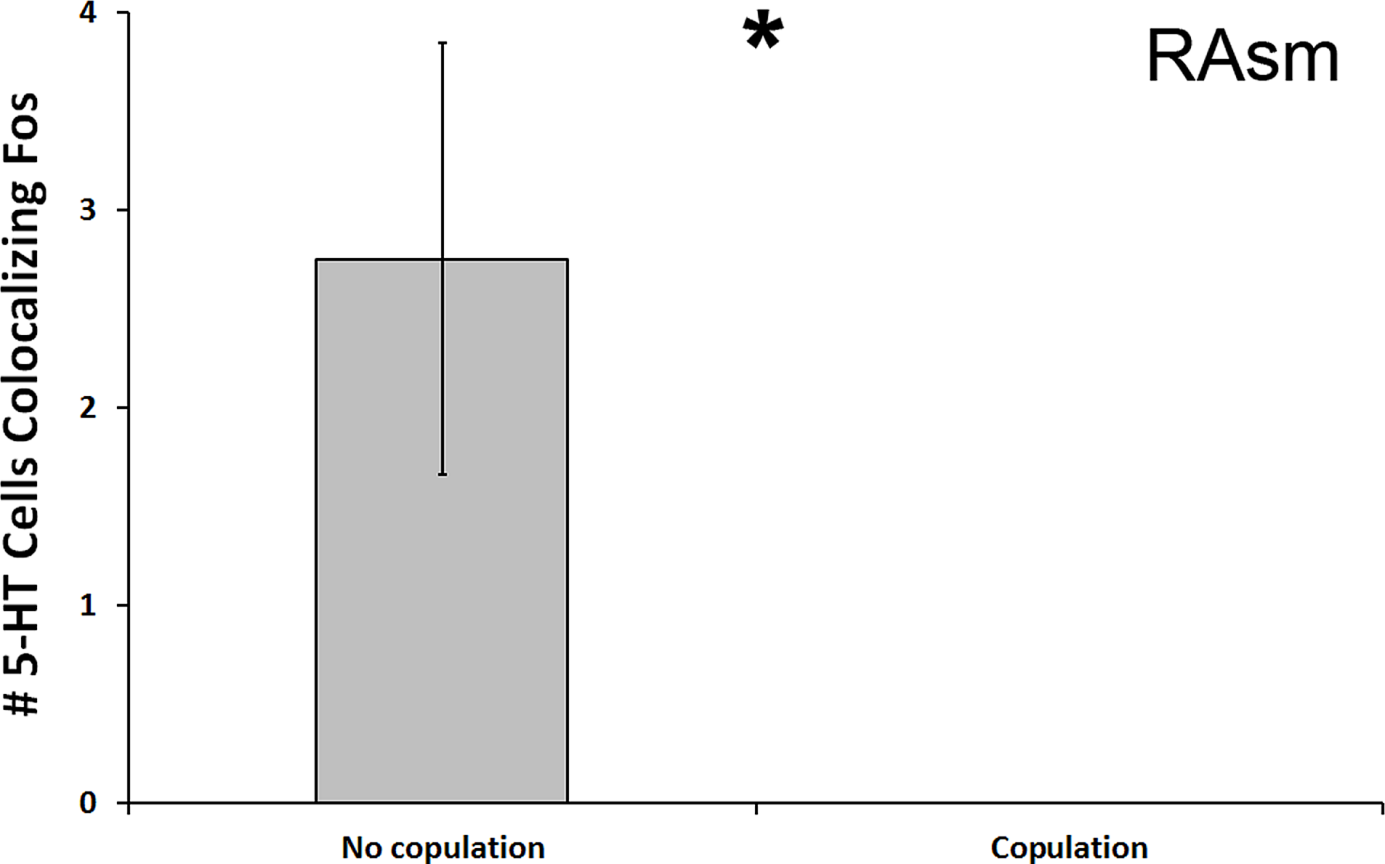
Fos induction in 5-HT neurons of copulators and non-copulators. A decrease in the total number of 5-HT neurons colocalizing Fos (throughout the whole nucleus) was seen in the RAsm of courtship subjects that copulated relative to those that did not copulate. (*) indicates a significant difference (*p* < 0.05) between behavioral intensity groupings.

We found a significant decrease in the number of 5-HT cells colocalizing Fos in the superior medial raphe nucleus (RAsm) of subjects whose courtship included actual copulation relative to those that did not copulate (*U* = 8.000, *N* = 20, *p* = 0.018; Fig. 4). Although these differences do not meet the threshold of the Benjamini-Hochburg adjusted α of 0.007, likely due to our small sample sizes, the difference between group is infinite as no copulating animal showed any 5-HT-Fos colocalized cells. Hence, we nevertheless report the results here for the reader’s benefit. This same effect was seen even if examining percent of 5-HT-Fos colocalization (*U* = 8.000, *N* = 20, *p* = 0.019). We saw no other differences between copulating and non-copulating animals in any other 5-HT population.

#### Aggression intensity (individual regions)

**Figure 5:**
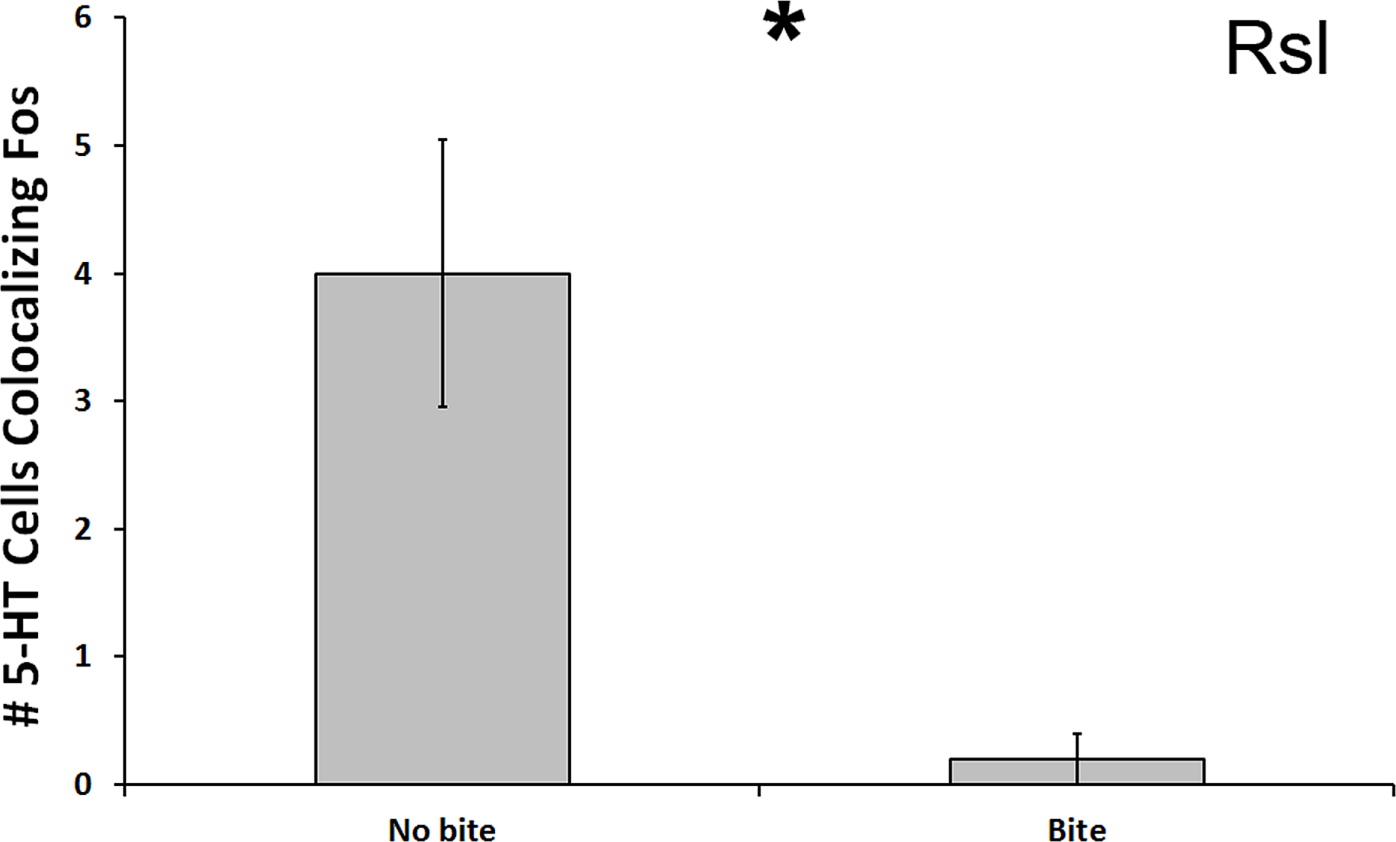
Fos induction in 5-HT neurons of biters and non-biters. A decrease in the total number of 5-HT neurons colocalizing Fos (throughout the whole nucleus) was seen in the Rsl of aggression subjects biting their opponents than in subjects that did not bite their opponent. (*) indicates a significant difference (*p* < 0.05) between behavioral intensity groupings.

In the Rsl, there was a significant reduction in number of 5-HT cells colocalized with Fos in subjects that bit the male conspecific relative to those that did not bite the conspecific (*U* = 13.500, *N* = 21, *p* = 0.024; Fig. 5). Although this significance level was not below the Benjamini-Hochburg adjusted α of 0.008, the difference between the group means was again great—this time 2000%. However, this time no significance was detected when examining percent colocalization (*U* = 16.500, *N* = 18, *p* = 0.089). We saw no other differences between biting and non-biting animals in any other 5-HT population.

### 5-HT correlations with behavior frequency and latency

When examining all 5-HT regions combined, there were no significant correlations between neural measures of 5-HT colocalization with Fos and behavioral frequencies or latencies in either courtship or aggression trials (*p* > 0.05 for all). However, correlations emerged when analyzing brain regions separately. Although none of these correlations resulted in a *p* value below the adjusted α for multiple comparisons, we nevertheless present them below as at least strong trends for the reader’s benefit, as they can serve to generate hypotheses for future studies.

#### Courtship behavior latency (individual regions)

**Figure 6:**
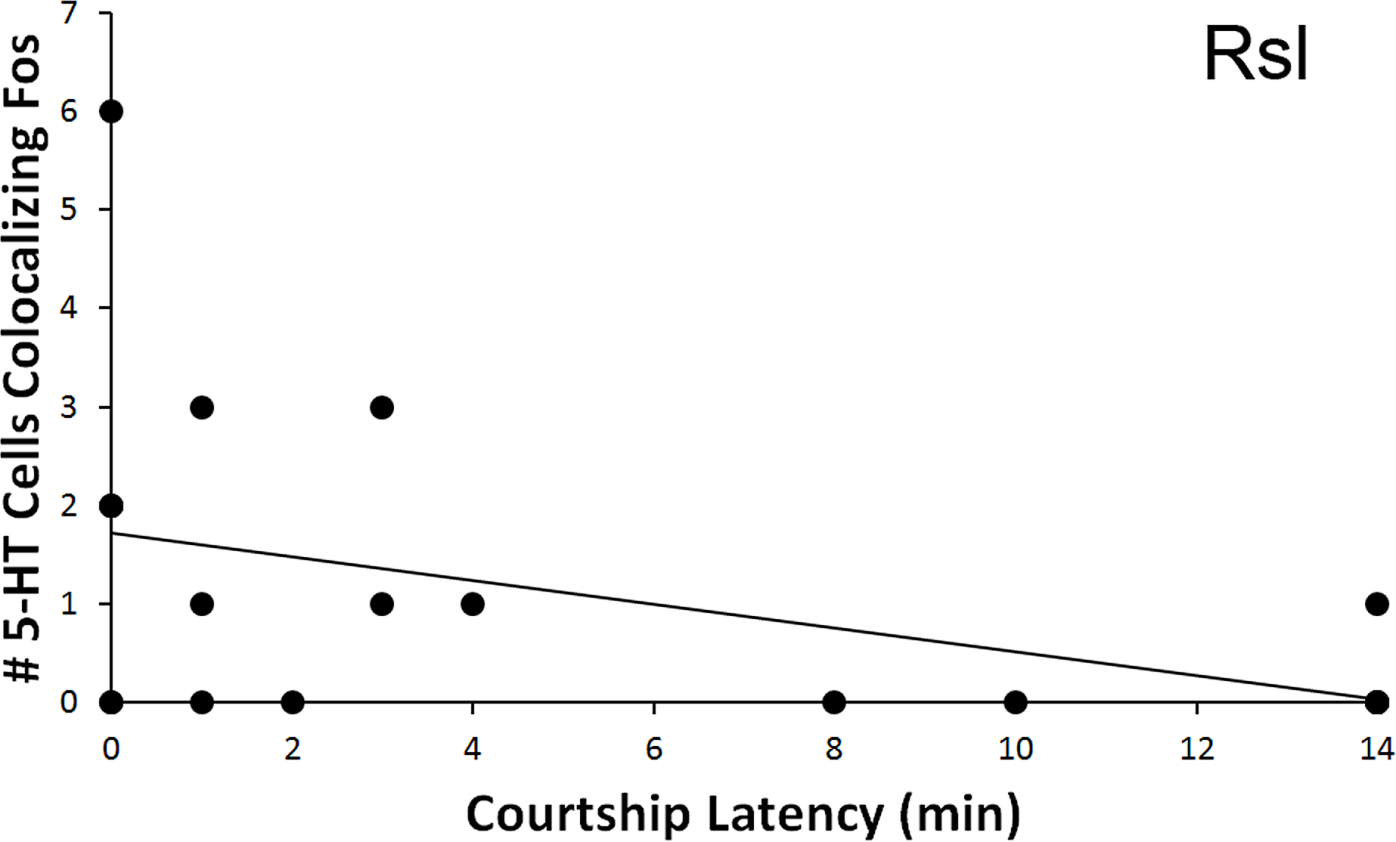
Fos induction in 5-HT neurons in relation to latency to display courtship behaviors. Spearman’s correlation (*r* =  − 0.467) between latency to courtship behavior and total number of 5-HT cells colocalizing Fos in the Rsl (*p* = 0.038).

The total number of 5-HT cells colocalizing Fos in the Rsl was found to correlate negatively with latency to first courtship display (*r* =  − 0.467, *N* = 20, *p* = 0.038, Fig. 6). No correlation was present when examining percent colocalization (*r* =  − 0.342, *N* = 21, *p* = 0.129). No other regions showed any significant correlations with behavioral frequency or latency (*p* > 0.05 for all).

#### Aggression behavior frequency (individual regions)

**Figure 7:**
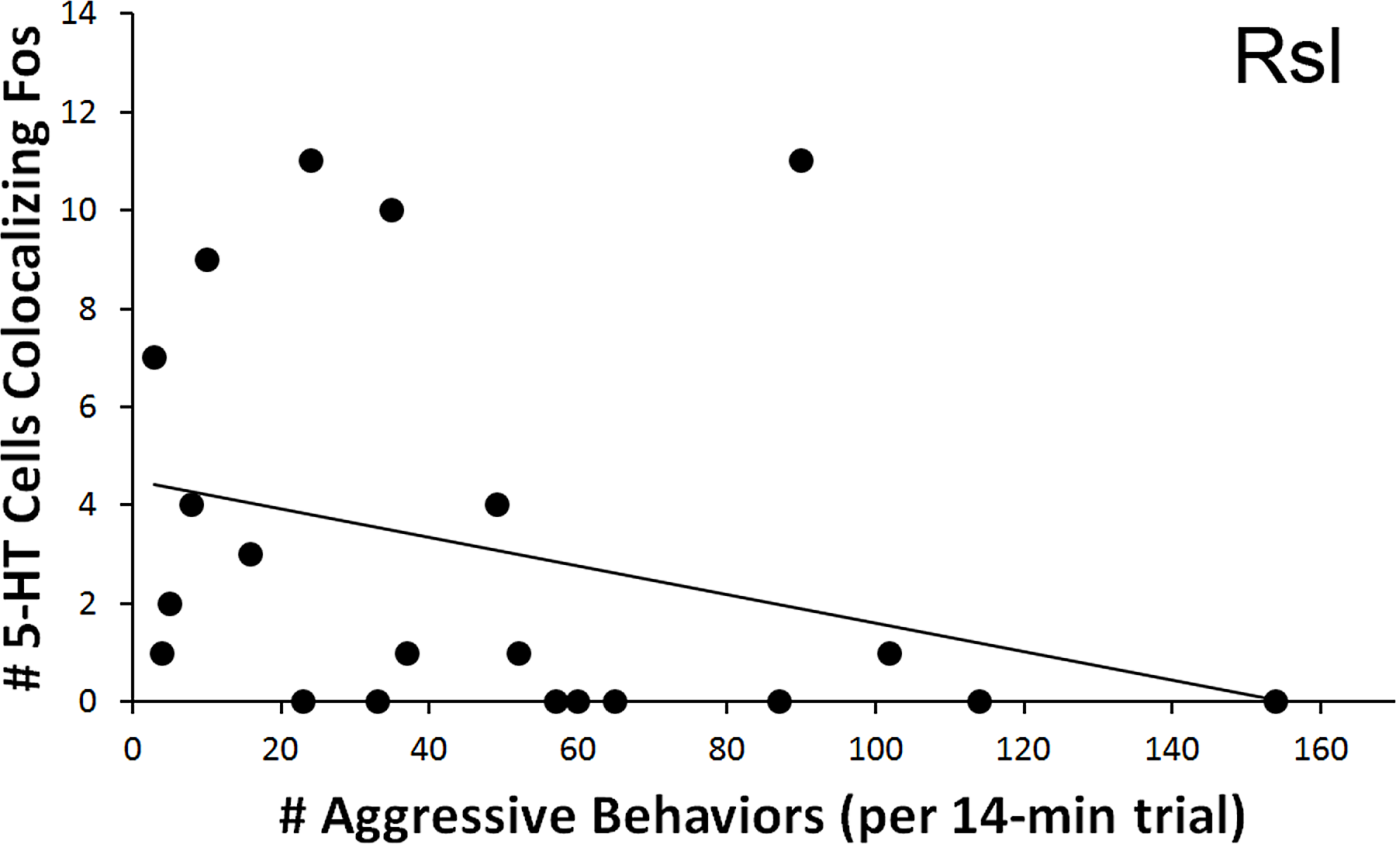
Fos induction in 5-HT neurons in relation to aggression frequency. Spearman’s correlation (*r* =  − 0.445) between aggression behavior frequency and total number of 5-HT cells colocalizing Fos in the Rsl (*p* = 0.043).

As with courtship latency, a significant relationship was found between behavioral measures and the count of 5-HT neurons colocalizing Fos within the Rsl. In relation to aggression, the total number of colocalized 5-HT neurons correlated negatively with the total number of exhibited aggressive behaviors (*r* =  − 0.445, *N* = 21, *p* = 0.043) (Fig. 7). No correlation was again found if examining percent colocalization (*r* =  − 0.374, *N* = 22, *p* = 0.086). No other regions showed significant correlations with aggressive behaviors.

## Discussion

The results of this study suggest that both aggression and courtship encounters lead to change in Fos-induction (activation) within 5-HT-producing cells in the raphe nuclei and superior reticular nucleus of the brown anole brainstem. These activity changes may be due to a combination of social environment and the subject’s own behavioral expression during social interaction trials.

When initially examining the effects of the social encounter condition on all 5-HT regions combined (a measure of total 5-HT output), only the courtship encounter was found to significantly alter (decrease) the number of 5-HT-Fos colocalized neurons relative to subjects in the control condition. However, when we conducted a more precise examination of the response to the social encounter condition of individual cell populations, we then found that both the courtship and aggression conditions altered 5-HT-Fos colocalization; these changes were seen in both the RAsl and the Rsm.

While the RAsl and Rsm were therefore found to be differently activated across type of encounter (a between-condition effect), we also detected within-condition relationships between exhibited behaviors and Fos induction within the RAsm and Rsl 5-HT neurons. First, strong relationships between neural activation and the intensity of focal animal behavior were detected, whereby a decrease in the total number of 5-HT cells colocalizing Fos in the RAsm and Rsl occurred specifically in subjects that responded to conspecifics with copulation (courtship encounter) or biting (aggression encounter), respectively, relative to those animals that only engaged in lower-intensity social behaviors. Second, the total number of 5-HT cells colocalizing Fos in the Rsl was negatively correlated with latency to first courtship behavioral display (i.e., positively correlated with quickness to display) by animals in the courtship condition. Third, the total number of 5-HT cells colocalizing Fos in the Rsl was also negatively correlated with the total number of aggressive behaviors performed by animals within the aggression condition. These findings suggest that, along with the effects of social encounter context (condition effect) on 5-HT cell activation, 5-HT activation is also linked, though via different cell populations, to measures of the behavioral response (intensity, frequency, and latency of behaviors within a condition). This relationship may be due to ties between the 5-HT system and circuits leading to motivation and motor output, or else due to differing perceptual inputs across individuals engaged in different intensities of social interaction, the intensity of which may vary between individuals due to other separate circuitries controlling behavioral output.

While the aggression findings in this study are generally consistent with previous research demonstrating inverse relationships between aggressive behavior and serotonin activity across multiple classes of vertebrates, this study suggests that courtship also partly shares this inverse relationship with serotonin activity (Conner et al., 2010; Audero et al., 2013; Zubizaretta et al., 2012; Bubak, Renner & Swallow, 2014). Furthermore, while most previous research has examined serotonergic release, our study examines changes at the source serotonergic cell populations. Therefore, complementing previous studies that measured general levels of serotonin output (Summers & Greenberg, 1995; Watt et al., 2007), the use of IHC in our study allowed for regional localization of effects to the RAsm, RAsl, Rsm, and Rsl–revealing differential localization of effects of social behavior encounter versus social behavior execution.

### Courtship

Source populations of 5-HT neurons that affect sexual behaviors include neurons in the raphe nuclei, which are suspected to regulate sexual behavior because lesions of the dorsal raphe nucleus (DRN) have been shown to decrease ejaculation latency in male rats as well as increase lordosis behavior in female rats (McIntosh & Barfield, 1984; Kakeyama, Negishi & Yamanouchi, 1997). The superior raphe nucleus, which we subdivide into medial (RAsm) and lateral (RAsl) parts, described in sharks and reptiles, is believed to be homologous to the mammalian DRN (Stuesse, Cruce & Northcutt, 1991; Kiehn, Rostrup & Møller, 1992). However, the lizard RAsm and RAsl do not appear to be as greatly differentiated into subnuclei as the DRN (Hale & Lowry, 2011) and the exact homology of the RAsm and RAsl to DRN subnuclei is unknown.

Furthermore, we identify the superior reticular nucleus, which we subdivide into medial (Rsm) and lateral (Rsl) parts based on spatial distribution and similar subdivision in other reptiles (Wolters et al., 1985; Smeets & Steinbusch, 1988; Challet et al., 1991; Kiehn, Rostrup & Møller, 1992). The reticular nucleus is found lateral to the superior raphe and believed by some to be homologous to the mammalian B9 nucleus (Stuesse, Cruce & Northcutt, 1991; Rodrigo-Angulo, Rodriguez-Veiga & Reinoso-Suárez, 2000). However, a separate pontomesencephalic reticular formation is also present in mammals (Hale & Lowry, 2011), and although the function of this region is not well understood, it does possesses projections to the basal forebrain. Both the B9 and the pontomesencephalic reticular formation show social behavior-related differences in gene expression (Kerman et al., 2011). Therefore, the functional associations to behavioral regulation seen in the present study (condition-dependent Fos induction in the Rsm and Fos induction related to aggression intensity and frequency, as well as courtship latency, in the Rsl) are consistent with homology with either or both of these two 5-HT groups in mammals.

Although not exhaustively explored, it has been suggested that the 5-HT system may influence courtship behaviors through connections to the bed nucleus of the stria terminalis (BNST) as well as through an interaction with the neuropeptide Y system (Finn & Yahr, 2005; Muroi & Ishil, 2016). Our study provides further support for the involvement of raphe nuclei in courtship behavior; however, we believe our study to also be unique as we know of no previous research that has linked 5-HT neurons of the reticular nucleus to courtship.

The mechanisms of 5-HT action at release sites pertinent to courtship behavior regulation are complex. Previous research has found that generally decreasing central 5-HT transmission facilitates copulatory behavior in rodents; however, 5-HT appears to have a complex regulatory role in which certain receptors activate, while others inhibit, sexual behaviors (Albinsson et al., 1996; Yonezawa et al., 2008; Carnevale et al., 2011; Uphouse, 2014). For example, 5HT _1*A*_receptors appear to facilitate ejaculation, while 5HT _1*B*_ receptors seem to inhibit copulatory behaviors (Fernandez-Guasti & Rodríguez-Manzo, 1992; Olivier et al., 2011). These functional differences of 5-HT based on receptor type and location could help explain our findings that a decrease in total 5-HT cells colocalizing Fos in the RAsm was found in animals that engaged in copulation, while the total number of 5-HT cells colocalizing Fos in the Rsl was nevertheless negatively correlated with latency to display courtship behavior. Projections of these two nuclei to different sets or types of post-synaptic receptors could explain the simultaneous positive relationship between courtship behaviors and neural activity in the Rsl and negative relationship with activationin the RAsm. Overall, the effects of 5-HT on sexual behaviors are still not fully understood and the effect of 5-HT on sexual behavior even appears to differ between sexes (Mariana & Kuhn, 2015).

Our study is also unique because it provides some evidence for 5-HT regulation of appetitive courtship behaviors—a correlation with latency to display of any courtship behavior (the first behavior displayed was always an appetitive display rather than the consummatory behavior of copulation). Such a link has not been well established. While 5-HT is known to primarily regulate consummatory behaviors (Dzieweczynski & Hebert, 2012; Fumero et al., 1994; Rhodes, Yu & Yamaguchi, 2007), it has been suggested that it is dopamine that plays a larger role in regulating appetitive behaviors. We have indeed confirmed the involvement of dopamine in appetitive (as well as consummatory) courtship behaviors in these same individuals within a separate study (Kabelik et al., 2014), but the present study suggests that 5-HT also has some connection to appetitive courtship display. However, the most striking results of our study are in accordance with the more well-established role of 5-HT in the regulation of consummatory behaviors, as we found a dramatic decrease in total 5-HT cells colocalizing Fos in the RAsm in animals that engaged in copulation compared to those that did not.

While most research focuses on the effects of 5-HT on social behaviors, relatively little research has examined how exposure to a social context can affect 5-HT activity. Past research along this line of inquiry has detected increased 5-HT levels in the striatum and nucleus accumbens of male rats after exposure to a female rat (Urakawa et al., 2014). An increase in 5-HT activity was also seen in the DRN of female salamanders when exposed to male pheromones (Laberge et al., 2008). Our between-group analysis adds to these findings by demonstrating the effects (decreased colocalization with Fos) of a courtship encounter on 5-HT activity regardless of the intensity or frequency of behavior elicited from the focal animal. These effects of social encounter condition were localized to the RAsl and Rsm, while two separate regions, the RAsm and Rsl, were linked to behavioral intensity and frequency—suggesting separate systems involved in perception of courtship context and in courtship behavioral expression. This finding is consistent with the idea of functional topography within subdivisions of midbrain and hindbrain serotonin-containing regions (Hale & Lowry, 2011; Commons, 2015). While the exact homology of the lizard subdivisions used in this study to mammalian subdivisions is not clear, the involvement of the more rostral regions (RAsl, Rsm, RAsm, and Rsl) and not the caudal regions (Rai and Raic) with courtship behavior found in this study is consistent with previous associations of rostral DR regions to reproductive behavior in mice (Commons, 2015).

### Aggression

Execution of aggressive behaviors has generally been associated with a decrease in 5-HT activity, although the opposite has also been reported (Korzan et al., 2001; Van der Vegt et al., 2003; Haller, Tóth & Halász, 2005; Audero et al., 2013). Our results extend this involvement of 5-HT in aggression to the brown anole and, as with the courtship condition above, suggest that some 5-HT populations respond primarily to social context, while the activity of others is associated with intensities and frequencies of behavioral expression.

As with the courtship analyses above, fewer 5-HT neurons colocalizing Fos were observed in the Rsm and RAsl of male brown anoles exposed to an aggressive encounter, relative to control subjects. In contrast, our within-group comparisons indicate a strong link between displayed aggression and 5-HT activity within the Rsl, whereby subjects exhibiting the most intense behavior, biting, showed dramatically lower levels of 5-HT cell activation than subjects exhibiting non-maximal levels of aggressive display. Activation of 5-HT cells in the Rsl also correlated negatively with the number of aggressive behaviors displayed (both appetitive and consummatory behaviors combined). These results strongly suggest that the Rsl is a nucleus associated with aggressive behavior expression, whereas the Rsm and RAsl are, as in the courtship analyses, regions responding to a perception of the social context.

Although 5-HT release is generally associated with inhibition of exhibited aggression, the source nuclei for this 5-HT release are not fully clear. For instance, our study found decreased 5-HT activity in the RAsl of aggression-group animals relative to control-group subjects. Aggressive encounters were instead found to *increase* 5-HT activity in the DRN of hamsters; however, this was only seen in the losers of the encounter (Cooper et al., 2009), suggesting other possible functions, and the increase may have been due to DRN subnuclei not homologous with the RAsl. Interestingly, rats exposed to aggressive visual and olfactory stimuli, but not an actual physical encounter, also showed an increase in raphe 5-HT activity (Haller, Tóth & Halász, 2005). Likewise, a study of green anoles found increased 5-HT activity in the raphe nuclei following an aggressive encounter (Korzan & Summers, 2004); however, this time the effect was only seen in dominant males. Hence, evidence clearly suggests an involvement of the superior raphe 5-HT population in aggression, but there may be contextual and/or species differences present in the relationship of these neurons to aggressive behavior display, and differential activation of neurons across specific raphe subnuclei may further complicate the situation.

Unlike the raphe nuclei, we found little research examining the role of the reticular nucleus in aggression, although the medial division of the reticular nucleus has been implicated in aggression in Siamese fighting fish, although not specifically through the 5-HT system (Gorlick, 1990). As mentioned previously, the reticular nucleus is not consistently defined across classes of vertebrate. Two potential mammalian homologs of the reptile reticular nuclei include the B9 nucleus and the pontomesencephalic reticular formation—and both of these nuclei showed changes in Fos expression in response to an intruder in rats (Kerman et al., 2011).

Overall, our findings for the superior raphe somewhat contradict previous literature (though already itself contradictory), while our findings in the reticular nucleus add to the sparse knowledge of the region’s role in social behavior regulation.

## Additional Information and Declarations

### Data Availability

The following information was supplied regarding data availability:
jhart257@gmail.com; alexisnsmith94@gmail.com; Kabelik, David (2016): Serotoninergic Activation during Courtship and Aggression in the Brown Anole. figshare.
https://doi.org/10.6084/m9.figshare.4477838.v1.
